# Bacterial Diversity and Community Structure of a Municipal Solid Waste Landfill: A Source of Lignocellulolytic Potential

**DOI:** 10.3390/life11060493

**Published:** 2021-05-28

**Authors:** Ogechukwu Bose Chukwuma, Mohd Rafatullah, Husnul Azan Tajarudin, Norli Ismail

**Affiliations:** School of Industrial Technology, Universiti Sains Malaysia, Penang 11800, Malaysia; ogechukwuma@student.usm.my (O.B.C.); azan@usm.my (H.A.T.); norlii@usm.my (N.I.)

**Keywords:** bacteria, biodiversity, landfill, lignocellulose biomass, lignocellulolytic enzyme, lignocellulolytic bacteria, metagenomics

## Abstract

Omics have given rise to research on sparsely studied microbial communities such as the landfill, lignocellulolytic microorganisms and enzymes. The bacterial diversity of Municipal Solid Waste sediments was determined using the illumina MiSeq system after DNA extraction and Polymerase chain reactions. Data analysis was used to determine the community’s richness, diversity, and correlation with environmental factors. Physicochemical studies revealed sites with mesophilic and thermophilic temperature ranges and a mixture of acidic and alkaline pH values. Temperature and moisture content showed the highest correlation with the bacteria community. The bacterial analysis of the community DNA revealed 357,030 effective sequences and 1891 operational taxonomic units (OTUs) assigned. Forty phyla were found, with the dominant phyla *Proteobacteria*, *Firmicutes*, *Actinobacteria*, and *Bacteroidota*, while *Aerococcus*, *Stenotrophomonas*, and *Sporosarcina* were the dominant species. PICRUSt provided insight on community’s metabolic function, which was narrowed down to search for lignocellulolytic enzymes’ function. Cellulase, xylanase, esterase, and peroxidase were gene functions inferred from the data. This article reports on the first phylogenetic analysis of the Pulau Burung landfill bacterial community. These results will help to improve the understanding of organisms dominant in the landfill and the corresponding enzymes that contribute to lignocellulose breakdown.

## 1. Introduction

Natural habitats contain a large variety of unstudied and yet-to-be-characterized microorganisms, which include unidentified bacteria communities. Much research states that microorganisms are still the biggest group of undescribed biodiversity and that they are a bewildering source of exploitable diversity [1]. Municipal solid waste (MSW) refers to waste coming out of homes and institutions, and over 2 billion tonnes are produced worldwide on an annual basis, with a 2050 projection of 3.4 billion tonnes [2]. The collection, management, and disposal of MSW are a huge environmental issue causing huge environmental problems such as air, water, soil, and esthetic pollution [3].

Landfills are currently the day-to-day method for disposing municipal solid waste, especially in most countries, Asian developing countries inclusive [4]. The heterogeneous nature of landfill sites means that they contain large amounts of lignocellulosic materials [5], whose inherent sugars are being viewed as forefront alternatives to fossil sources in meeting industrial demands [6]. Lignocellulose biomass consists mainly of cellulose and hemicellulose bound together by lignin. It is the most available biopolymer on the Earth, which, in turn, accounts for lignin and cellulose being the most abundant polymers [7,8]. Any biomass that contains cellulose and hemicellulose as its major component can theoretically be used as a substrate in the generation of bioenergy [9].

Microorganisms via enzymatic breakdown of lignocellulose lead to the production of fermentable sugars used to make bioethanol among other products [10]. They are also crucial in the decomposing of organic waste as the availability of organic waste in MSW landfills makes the landfill a rich community of microbes [4,11]. Lignocellulolytic enzymes, biocatalysts also called lignocellulases, include hydrolytic enzymes that break down lignocellulose into simpler forms for further use [12]. Thus, a more in-depth understanding of the role of microorganisms is needed, particularly bacteria in the landfill. This will shed more light on stabilization of solid wastes and act as useful indicators for better planning and future management [13].

Bacteria display a wide diversity of function, with studies showing they grow more when there is more lignin and complex carbon present, thus making them a likely contributor for future biotechnology applications [8]. It is well documented that culture-dependent approaches create bias in the study of microbial diversity, which can be overcome by metagenomics’ studies, which involve amplifying phylogenetic marker genes to discover unknown and unculturable microorganisms [14]. Culture-dependent methods are unable to fully capture the amount of bacterial diversity of lignin degraders, making the grasp of the function and ecology of these communities minimal at best. This is a challenge, as removal of lignin is key in any conversion or deployment of lignocellulose biomass to useful end products [15].

Metagenomics gives information on the metabolic and functional diversity using genomic DNA extracted directly from environmental samples rather than a pure culture. A study of two landfills in China found that sampling site rather than sampling depth affected the makeup of the bacteria community, which had Firmicutes, Bacteroidetes, and Proteobacteria as the most abundant phyla [16]. Microbial succession and diversity in composting was studied and the dominant species recorded are known to release lignocellulolytic enzymes largely responsible for organic matter decomposition [17]. Cellulolytic potential of isolates from a landfill was studied and the reports showed cellulolytic species that were able to adapt to both mesophilic and thermophilic temperatures [18]. This study focused on the prokaryotic diversity in the landfill to shed more light on how these communities operate in the ecosystem. It also provided an inventory of bacterial consortia and dominant species, giving useful information regarding the microbial diversity of the Pulau Burung landfill. Subsequently, this information can be mined for biotechnological purposes. The objectives of this study were (1) to document the bacterial community in Pulau Burung Landfill Penang and (2) to identify the most prevalent bacterial groups that could be of biotechnological importance via their involvement in degradation of lignocellulosic waste.

## 2. Materials and Methods

### 2.1. Study Sites and Sampling

**Figure 1:**
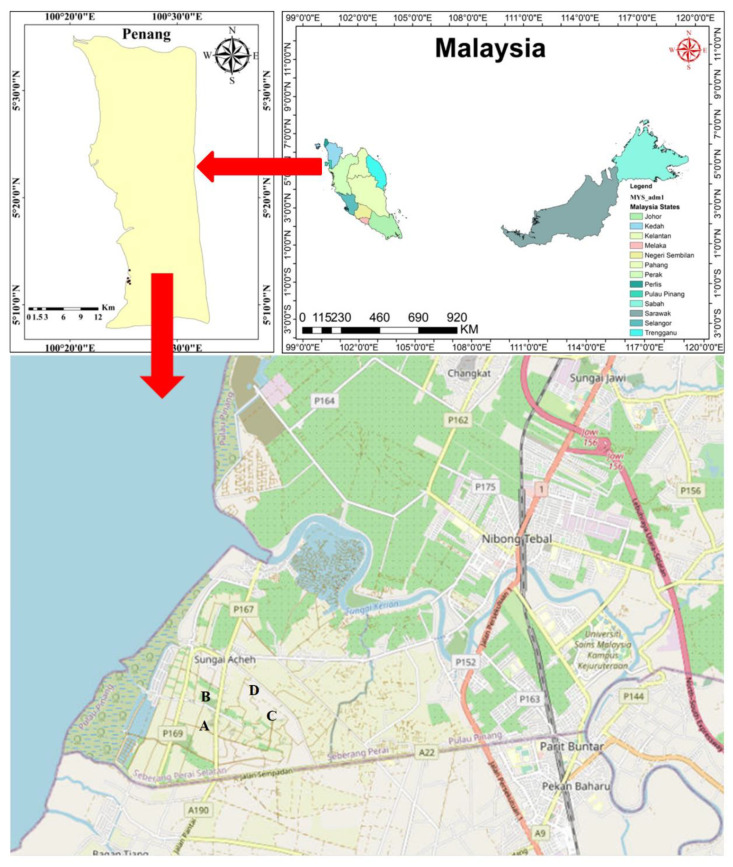
Location of study sites and layout of the sampling points in Pulau Burung Landfill, Penang, Malaysia. (Top right—Map of Malaysia; Top left—Map of Penang; Bottom—Study area detailing the sampling points in the landfill.

Pulau Burung landfill is a semi-aerobic, municipal landfill located in Nibong Tebal Penang, Malaysia. The sampling points are located at 5°11′ N; 100°25′ E (Figure 1). The landfill began its operations in the early 1980s and is now privately managed [19]. Sediment samples were collected from MSW cells in the landfill, using random sampling. Samples A and B were obtained from waste that was deposited 30 days prior, while C and D were gotten from freshly deposited waste less than 7 days prior to sampling. Coordinates and temperature were taken immediately. The samples were obtained in triplicates and then transported to the laboratory for further physicochemical and bacterial diversity studies. They were labelled A–D.

### 2.2. Physicochemical Properties

Physiochemical analysis was carried out with air-dried sediment samples. Moisture content was determined gravimetrically while, for pH measurements, soil–water (1:5, *w*/*v*) suspension was prepared and allowed to stand for 30 min before taking measurement with a pH electrode, which was attached to a pH meter (Mettler-Toledo, Zurich Switzerland). The total carbon (TC), total hydrogen (TH), and total nitrogen (TN) were tested by an elemental analyzer.

### 2.3. DNA Extraction and Sequencing

Genomic DNA in samples was extracted using GF-1 Soil Sample DNA Extraction Kit (Vivantis, Selangor, Malaysia). The extraction and subsequent analysis was done in triplicates following the manufacturer’s instructions. The quality of the purified DNA was first monitored on 1% TAE agarose gel. The concentration of DNA was measured using spectrophotometer (Implen NanoPhotometer^®^ N60/N50, Munich, Germany) and fluorometric quantification using iQuant^™^ Broad Range dsDNA Quantification Kit (Rockville, MD, USA). These protocols were done as previously described by Sinclair et al. [20]. Aliquots of 1 μL Genomic DNA (gDNA) were run on 1% TAE agarose gel at 100 V for 60 min.

### 2.4. PCR Amplification and Sequencing

The purified gDNA passed through DNA QC, and then amplification was carried out using locus-specific sequence primers to amplify bacterial 16s V3–V4 regions (forward primer, CCTACGGGNGGCWGCAG, and reverse primer, GACTACHVGGGTATCTAATCC) [20]. The PCR reactions were carried out with REDiant 2X PCR Master Mix (1st base) with the PCR protocol in the thermal cycler at 95 °C for 3 min, followed by 25 cycles at 95 °C for 30 s, 55 °C for 30 s, and 72 °C for 30 s, and a final extension at 72 °C for 5 min. It was then held at 4 °C. The 16S V3–V4 bacterial regions were amplified using locus-specific sequence primers. The forward primer used was TCGTCGGCAGCGTCAGATGTGTATAAGAGACAG and the reverse was GTCTCGTGGGCTCGGAGATGTGTATAAGAGACAG [20]. The PCR reactions (2nd base) were carried out using KOD -Multi and Epi-^®^ (Toyobo, Osaka, Japan). Dual indices were attached to the amplicon PCR using Illumina Nextera XT Index Kit v2 following manufacturer’s protocols. The quality of the libraries was measured using Agilent Bio-analyzer 2100 System by Agilent DNA 1000 Kit and fluorometric quantification by Helixyte GreenTM Quantifying Reagent.

### 2.5. Next-Generation Sequencing and Data Analysis

The libraries were normalized and pooled according to the protocol recommended by illumina and we proceeded to sequencing using MiSeq platform using 300 PE. Paired-end reads were merged using FastQC, and then quality was assessed to remove any below a quality score of 20 and reads < 150 bp. Data processing was done as described previously in Sinclair et al. [20]. The filtering and trimming of forward and reverse adaptors from the sequences, which are quality control steps, were carried out. Phylogenetic affiliations of the 16S rRNA gene sequences were done with RDP Classifier software and SILVA (SSU119) 16S rRNA database. Statistical analysis was performed using the VEGAN package and the R statistical framework versions 2.0 and 2.11, respectively. This included the diversity indices, Venn diagram, and correlation analyses. The principal component analysis (PCA) based on weighted UniFrac distance was done, and the taxonomic classification down to the phylum, class, and genus levels was carried out. Microbial community richness (Ace and Chao) and alpha diversity (Shannon and Simpson estimators) indices were assessed. The QIIME program was used to derive this while functional profiling of the catabolic genes in the individual metagenomes was predicted through PICRUSt (Phylogenetic Investigation of Communities by Reconstruction of Unobserved States) including the 16S copy number abundance and prediction of functional enzymes, as previously described by Wang et al. and Wongwilaiwalin et al. [21,22]. The bioinformatic pipelines are summarized in Figure S1. Unless otherwise stated, the mean values for analyzed results are captured in the results.

## 3. Results

### 3.1. Physicochemical Characteristics

The results of the physicochemical analysis are shown in Table 1 below. Samples A and D had the highest moisture content. Temperature was mostly mesophilic except for sample B that was thermophilic at 45 °C. The pH was acidic for samples A and B but alkaline for C and D. Samples B and D had the highest organic content for all three elements analyzed, while C showed the lowest.

### 3.2. Bacterial Analysis

A total of 357,030 effective sequences with average length of 455.76 bp were obtained and the minimum number of effective sequences was 80,189. The sequence reads were used to examine how diverse the bacteria community was between samples. The sequencing coverage rate exceeded 99% and a total of 1891 OTUs were assigned. An average of 847 ± 310 OTUs was identified with the minimum values of 558 being observed in Sample B. The Alpha diversity of the samples was also analyzed to reveal microbial community evenness and richness and is captured in Table 2.

The Chao and Ace indices were determined to express the richness of the microbial community. The average Chao and Ace index values for the bacterial community were 833 and 834. The Shannon and Simpson indices were also derived and had average values of 4.2 and 0.94. The richness and diversity of the landfill’s bacterial community are shown in Table 2. The Ace and Chao indices are used to capture richness in a bacterial community. Samples A and B had lower averages of Ace and Chao index values while Samples C and D had higher values, which indicates higher microbial richness in Samples C and D. Simpson and Shannon indices show the trend of diversity. The higher the Simpson value, the lower the microbial diversity, while the Shannon values’ higher values connote higher diversity. Sample D, again, had the lowest average Simpson value and one of the highest Shannon value. This implied that community from Sample D was the richest and most diverse of the samples analyzed. Samples C and D showed a higher diversity and this could be linked to the age of the waste in the landfill, as they were both from waste that was a week old in the landfill, implying that the sequencing depth was appropriate for a detailed description of the overall bacterial community. The rare fraction curve was determined using the bacterial OTUs as a means of looking at sampling saturation (Figure S2). The curves showed that the samples from Samples C and D showed a higher number of OTUs (indicating higher diversity). The rarefaction curves in Figure S2 attained a plateau stage for all the samples, which indicates that the depth of sequencing was appropriate to capture the general bacterial community.

**Figure 2:**
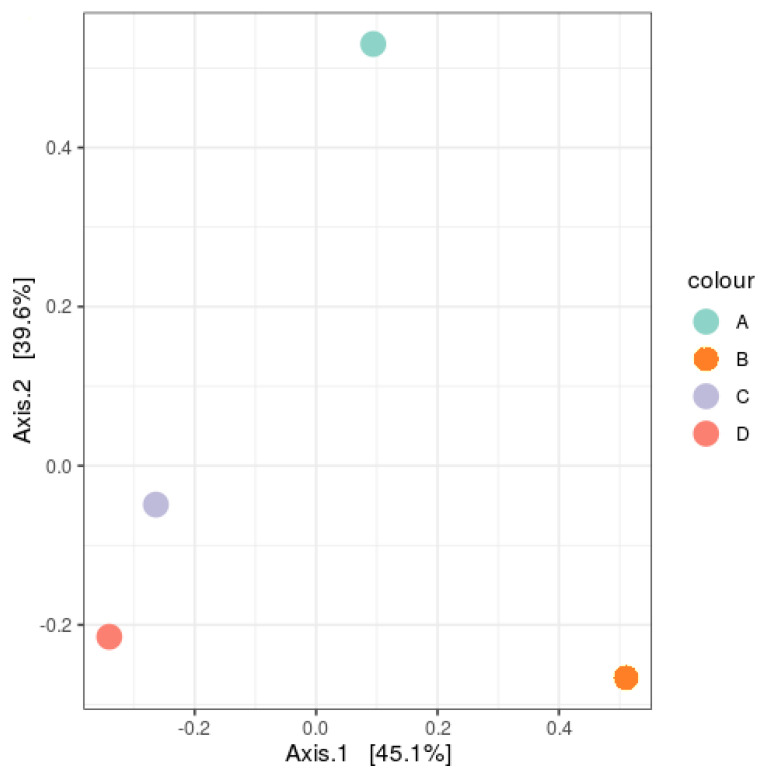
Principal coordinates analysis (PCoA) of the bacterial community structure showing plot based on the similarity coefficients of bacterial communities. Points closer to one another in ordination space indicate a closer similarity than those farther apart. Axes 1 and 2 represent principal components 1 and 2, 45.1% and 39.6% of the total variations, respectively.

**Figure 3:**
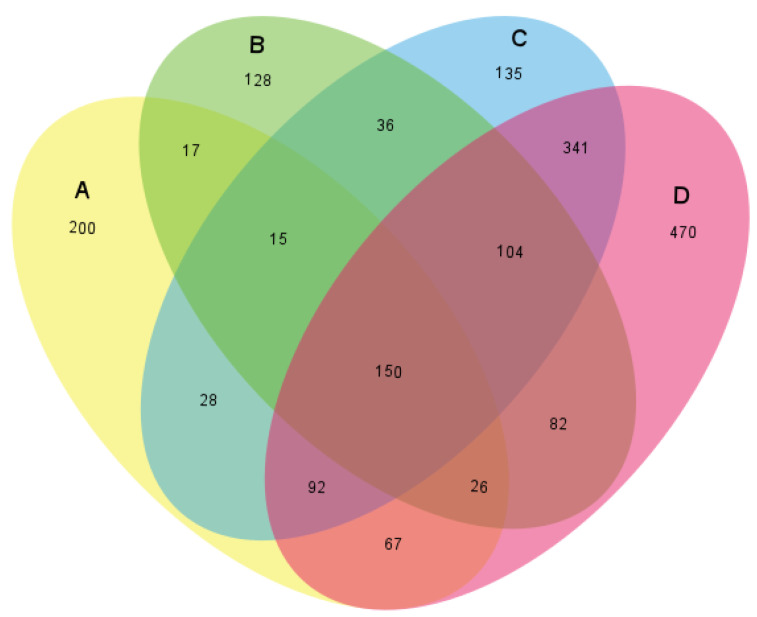
Observing similarities among the samples. The Venn diagram showing the number of shared and unique OTUs between the libraries of bacterial 16S rRNA genes on sediment samples A to D.

Principal component analysis (PCA) plots were analyzed to see the variance of the community, and a Venn diagram was prepared to capture the distinct and similar OTUs in the landfill samples. The principal component analysis plots (Figure 2) showed C and D fell to the left of the graph along the second principal component (Axis 2), while A was found in the upper parts of the middle of the graph. B was grouped to the right of Axis 1 and accounted for 45.1% of the entire variations. The C and D samples were grouped into a cluster, while A sample showed a distinct separation from B at the upper side of the graph along Axis 2. This stood for 39.6% of the entire variations and was an indication of an entirely different community structure. The total variations were 84.7% in both principal components of the different communities, as the samples tended to show three clusters, with C and D being closer and most similar. These values from the PCA tallies with the analysis from the Venn diagram. Sample C and D shared the highest number of OTUs, 341, which was unique to just both of them. An analysis of the Venn diagram (Figure 3) showed the number of common species within the prepared libraries. The four samples shared 150 OTUs, and 49% of the OTUs were seen in only one sample. The result also showed that Samples D and A had the highest number of unique OTUs (470 and 200, respectively), which meant they were the most distinct, While Samples B and C had the least (128 and 135, respectively).

### 3.3. Bacterial Taxonomy Composition

**Figure 4:**
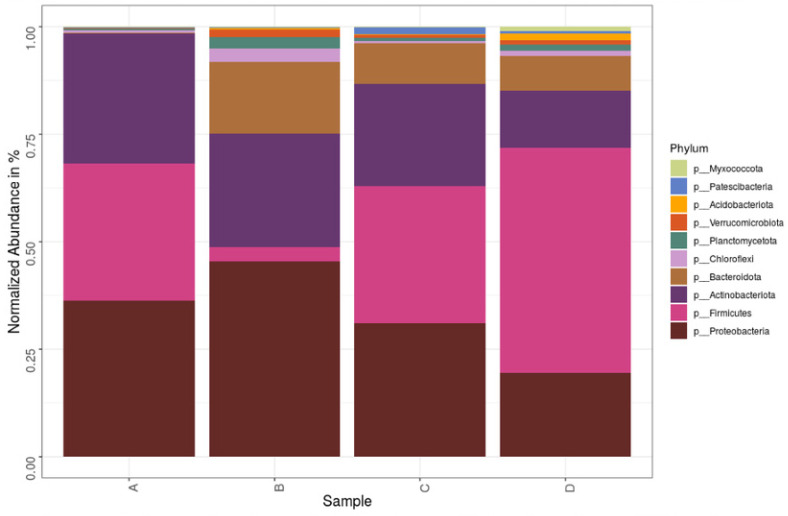
Composition of the bacterial community in MSW sediments from the landfill at the phylum level.

**Figure 5:**
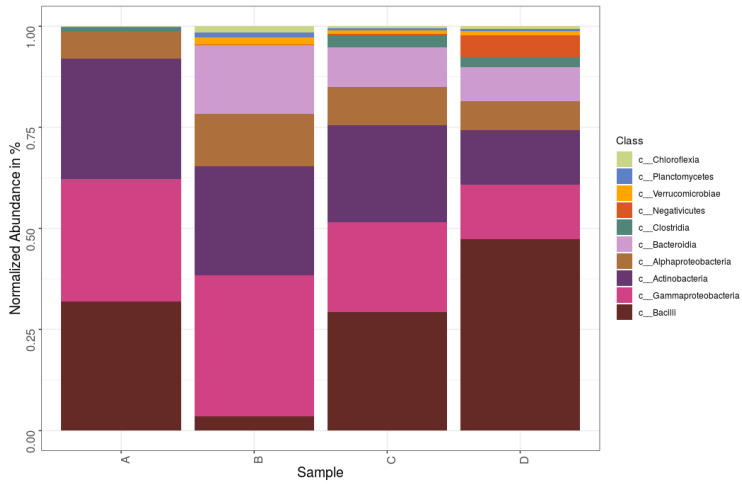
Composition of bacteria in MSW sediments from the Pulau Burung landfill at the class level.

**Figure 6:**
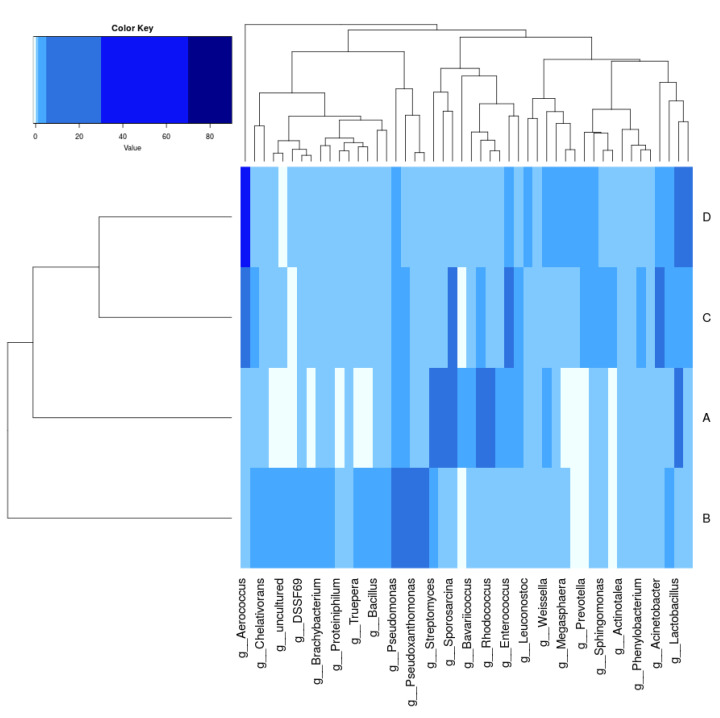
Heatmap of bacterial distribution of from the four samples at the genus level. Row represents the relative percentage of each bacterial genus, and column stands for different samples. The relative abundance for each bacterial genus was depicted by color intensity with the legend indicated at the top of the figure.

In total, 40 phyla were found and four phyla, *Proteobacteria*, *Firmicutes*, *Actinobacteria*, and *Bacteroidetes*, were the most dominant in all samples. Figure 4 shows the top phyla identified in each of the samples. At a glance, it is seen that the diversity of samples B, C, and D was higher than A. Sample A community had over 90% of its community from either the phyla *Proteobacteria*, *Firmicutes*, or *Actinobacteria*, while for samples B, C, and D it was *Proteobacteria*, *Firmicutes*, *Actinobacteria*, and *Bacteroidetes*, with *Bacteroidetes* exceeding *Firmicutes* in Sample B. *Bacilli* and *Gammaproteobacteria* were the dominant classes (Figure 5), with the major classes coming from *Actinobacteria*, *Proteobacteria*, *Bacteroidetes*, and *Firmicutes*. However, *Verrucomicrobiae*, *Chloroflexia*, and *Planctomycetes* also contributed to the 10 major classes in Samples B, C, and D but were absent Sample A. At the family level, 377 different families were captured, while on the genus level there were 708 genera. The top genera included *Aerococcus*, *Pseudomonas*, *Stenotrophomonas*, *Sporosarcina*, and *Lactobacillus*. The heat map analysis of the data was done to give the bacterial relationship of the community at the genus level. Figure 6 captures this microbial community structure with a heat map that shows high and low abundance in each sample. The legend at the upper, left corner shows the relative abundance for each bacterial genus distinguished by color intensity. Findings showed that the majority of sequences belonged to *Aerococcus* (8.60%–10.75%) and *Pseudomonas* (5.3%–6.95%) and were present in all samples. Sequences belonging to *Prevotella*, *Veillonella*, and *Proteiniclasticum* were found in D and C but absent in A and B, while *Actinotalea* was found in A and B but absent in C and D. The bacterial genera *Pseudomonas*, *Aerococcus*, *Lactobacillus*, and *Pseudoxanthomonas* were most abundant in D when compared to others, while *Bacillus* and *Leuconostoc*, from the color distribution observed, were evenly distributed among all the samples. Table S1 lists the top species and their relative abundance. Sample B had the highest occurrence of Proteobacteria but lowest of Firmicutes. Sample A had the highest incidence of Actinobacteria when compared to the other samples but the lowest amount of Bacteroidota. Sample C had the highest amount of Patescibacteria. Sample D had the highest Firmicutes and Acidobacteria.

### 3.4. Correlation between Bacterial Communities and Soil Physicochemical Properties

**Figure 7:**
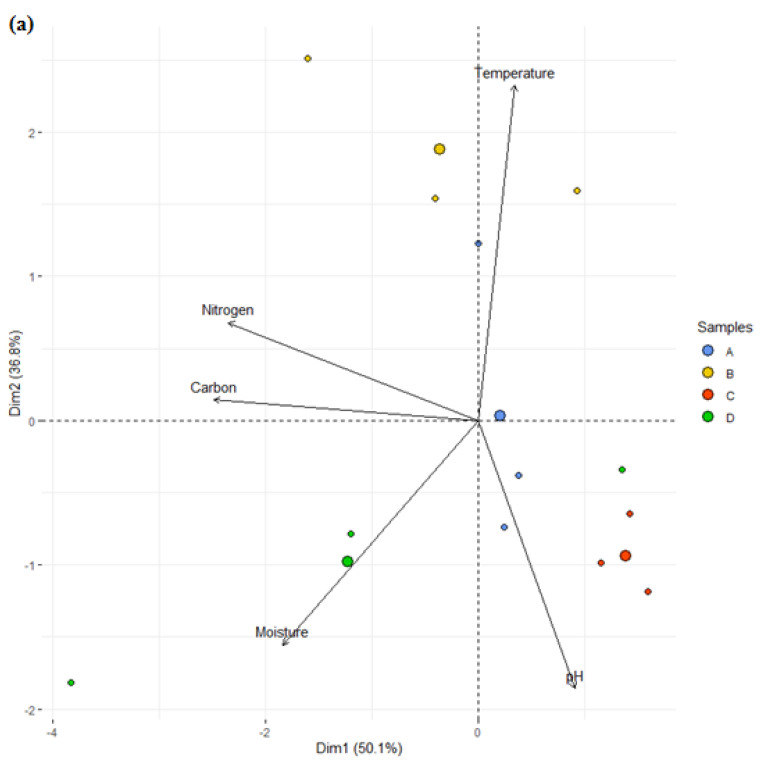

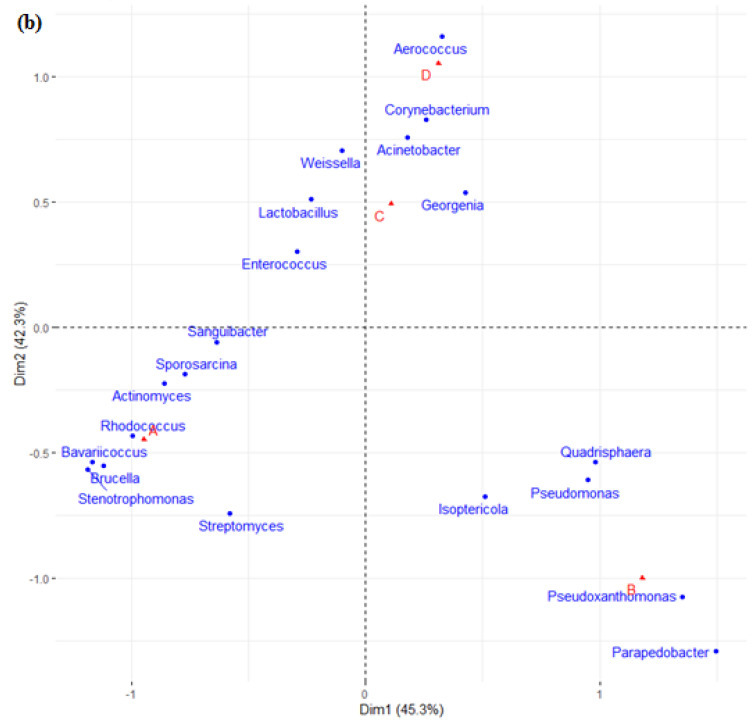
Correlation analysis showing relationship between (**a**) the bacterial community and the physicochemical properties (**b**), the distribution of the top genus observed in the bacterial community.

Figure 7 shows the correlation between the physicochemical properties and the bacterial community, which was analyzed using R correspondence analysis (R software Bi-plot analysis). The correspondence analysis showed that only carbon and nitrogen showed correlation among the physicochemical analysis. Moisture and pH explained 50.1% of the bacterial communities while carbon, nitrogen, and temperature explained 36.8%. The samples from A and B were also shown to make up 45.3% of the top genus, while that from C and D made up for 42.3%. Temperature and moisture had *p* values of *p* = 0.023 and *p* = 0.024, respectively, while carbon and nitrogen had *p* ≥ 0.05. The samples with the same aging characteristic were observed to be closer than those with different aging characteristic.

### 3.5. Function Prediction of Bacterial Communities

**Figure 8:**
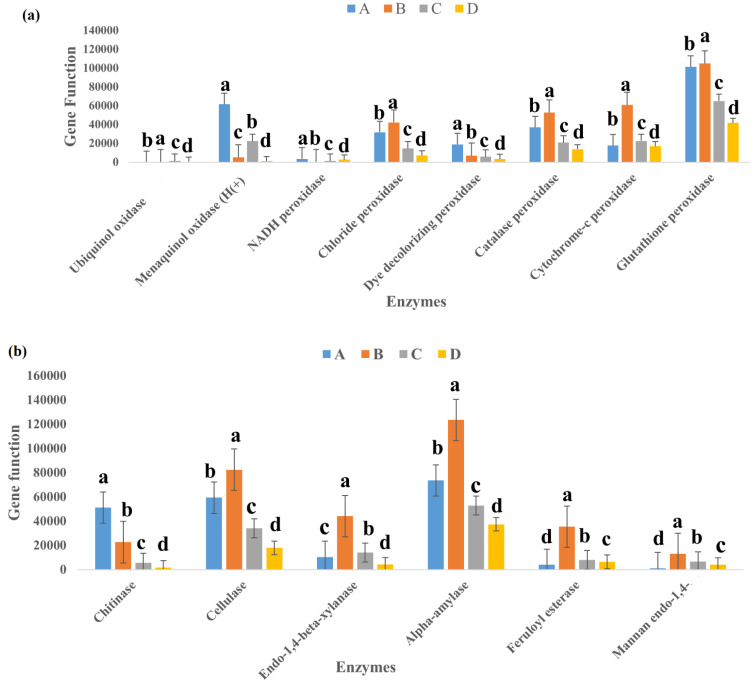
Summary of prediction of lignocellulolytic enzyme gene functions, as seen in Samples A–D. (**a**) Ligninolytic family of enzymes, showing their function inference at each site. (**b**) Cellulolytic enzymes inferred in the different samples. Bars represent standard errors. Different letters over columns indicate significant differences between various samples.

PICRUST predicts and compares probable functions in various habitats from available 16S data [23]. By using PICRUSt, KEGG pathway functions were used for functional annotation of the 16S rRNA-based metagenome [24]. The Enzyme Commission (EC) report was generated for the entire community and had 2300 identified ECs. The EC number is particular to an enzyme as it is assigned considering the chemical reactions it catalyzes [25]. The report showed high presence of class EC 1-Oxidoreductases (629) compared to EC 3 Hydrolases (499). The major lignocellulolytic enzymes are laccase, peroxidase, cellulase, xylanase, amylase, chitinases, esterase, and mannanase [12]. Only Laccase, Pectinase, and Protease were not specifically identified with their EC numbers, as seen in Figure 8a,b. Samples A and B showed higher functional inference for Cellulase, Peroxidase, Amylase, Chitinase, and Xylanase, while the enzymes amylase, cellulase, and peroxidase had the functional inference in all the samples. Sample D, on the other hand, had the lowest values for all lignocellulolytic enzymes considered. Statistical analysis showed that there was significant difference for all the enzymes in each of the samples.

## 4. Discussion

It has been established that culture-independent techniques need to be employed to boost culture-dependent techniques, which are limited in output, to garner more information on microbial communities and their possible function in the environment [26]. Bacteria, especially those with lignocellulolytic abilities, hold enormous potential for application in biotechnology [4,5,27,28].

In the physicochemical study, Samples D and C had the highest pH but the lowest temperature. Sample B, despite having the highest temperature, had one of the lowest pH. Sample D had the highest moisture, carbon, and hydrogen content. The pH and moisture content values, when compared to those from previous studies, as reviewed by Sekhohola-Dlamini and Tekere [29], were within the ranges for active older landfills. Moisture content and pH are important in the processes of hydrolysis, fermentation, and bioavailability, as they help regulate microbial respiration [29]. The correspondence analysis (Figure 7) showed that temperature had the highest effect on the bacterial community distribution, alongside moisture and pH. Other studies have also shown that temperature and moisture are prominent factors in determining microbial community structure [4]. Moisture content showed positive correlation with pH, while it had a negative correlation with temperature. Similar studies by Wong et al. [30] stated that bacterial communities were significantly (*p* < 0.05) affected by moisture content, organic matter, and pH. These results are similar to the results from our physicochemical studies.

Sample B, which was slightly thermophilic, had the most diverse class of bacteria at the class level, which contained Bacteroidia with an established link to conveniently break down complex and recalcitrant biopolymers [31]. Sample B also contained the largest proportion of Chloroflexi, a characteristic thermophilic organism previously reported in a hydrothermal vent [32]. Organic matter influences the available substrate needed as energy source by living things and is a key factor that shapes microbial community. Studies show that high nitrogen content of food waste can lead to an accumulation of ammonia. This can inhibit microbes primarily involved in anaerobic digestion, especially the methanogens. Low nitrogen content, as seen in this study, implies that the environment will be favorable to anaerobic digestion. Carbon is essential for growth in microbial communities and, in turn, the structure and diversity of the bacterial community [33], but it showed the least correlation to the distribution of the bacterial community.

When looking at the OTUs observed in each sample, the sequence was Sample D > C > A > B. Each of them showed significant difference to each other. Microbial richness (Chao1 and Ace) followed the same sequence as observed OTUs. The richness of each of the samples was significantly different from one another. Alpha diversity indices Shannon and Simpson had a different sequence, as Sample C > D > B > A. For Shannon indices, there was no significant difference between C and D, which had the same aging characteristic in the landfill. However, there was significant difference between them and samples A and B. For Simpson values, there was no significant difference between Samples A, B, and D. Moreover, there was no significant difference between Samples C and B, while C was different from A and D.

The dominant phyla in this study were *Proteobacteria*, *Firmicutes*, *Actinobacteria*, and *Bacteroidetes*. They made up 32.5%, 29.5%, 23%, and 8.5%, respectively, of the total bacteria community, which accounted for over 90%. They are known to be top phyla in highly diverse bacterial communities [34]. They are identified lignocellulose biomass degraders in several communities ranging from peat forests to switch grass [35,36]. These phyla are also dominant groups in composting. Studies of microbial consortia with *Actinobacteria* reported it as the top lignocellulolytic phylum and *Firmicutes*, known to only degrade the simpler organic compounds [37,38]. In this study, Firmicutes had a higher incidence in Samples C and D, which were from waste that was about a week old in the landfill, while Proteobacteria had a higher incidence in Samples A and B, which were 30 days old. Bacterial communities vary in composition as succession occurs in various stages of solid waste decomposition [39].

*Actinobacteria*, *Firmicutes*, *Proteobacteria*, and *Bacteroidetes* are garnering attention as regards to application in bioconversion industries because they grow rapidly on laboratory media and have shown great promise and efficiency in biodegradation of biomass [40]. *Firmicutes* are the most dominant phylum, as seen in other studies where it was most dominant in landfill studies. They are cellulose-degrading bacteria thought to be involved in anaerobic and methanogenic breakdown of waste in landfills [41]. *Chloroflexia*, *Planctomycetes*, and *Verrucomicrobia* were other phyla in the bacterial community, and they accounted for 1.2% of the total community. They are cellulolytic groups known to be cultivation-resistant phyla [15]. *Patescibacteria* was reported in anal droplet of insect in a community still dominated by *Proteobacteria*, *Firmicutes*, *Actinobacteria*, and *Bacteroidetes* [42], suggesting an unclear synergistic relationship between these groups. *Proteobacteria*, *Firmicutes*, and *Actinomycetes* are already known to produce peroxidases, which require oxygen to perform their function [7].

*Bacilli*, *Gammaproteobacteria*, *Actinomycetes*, and *Alphaproteobacteria* were the dominant classes in the landfill samples. These results are similar to previous studies on taxonomic composition of bacteria in landfills at the class level, as reviewed by Sekhohola-Dlamini and Tekere [29]. Some bacteria are already known to break down lignin, and most come from the *Alphaproteobacteria*, *Gammaproteobacteria*, and *Actinomycetes* classes [43]. *Aerococcus*, *Pseudomonas*, *Stenotrophomonas*, *Sporosarcina*, and *Lactobacillus* were the dominant genera. *Pseudomonas* has been previously reported as a dominant genus by Heo et al. [44]. However, *Aerococcus*, which was classified as a dominant airborne genera by Puentes-Téllez and Falcao Salles [45], and *Sporosarcina* are deviations from other landfill studies. *Stenotrophomonas* sp., more common in the rhizosphere of plants, is known for its laccase potential, which leads to breakdown of lignin [46]. Our study also showed *Myxococcus* as a top genus, which was previously reported when the pill bug *Armadillidium vulgare* was studied by Ventorino et al. [47] for lignocellulose-degrading function. It was reported as a key contributor to reported genes for encoding lignocellulose-binding modules. *Streptomyces* is also a top genera and plays a dominant role when industrial microorganisms are looked at. It is presently the richest known source of bioactive molecules (enzymes) and is, thus, an interesting subject for industrial considerations as it has potential for green applications in producing chemicals, biopharmaceuticals, and biofuel production [48].

The most dominant species were *Aerococcus urinaeequi*, *Stenotrophomonas rhizophila*, *Sporosarcina_psychrophila*, *Pseudoxanthomonas taiwanensis*, and *Corynebacterium vitaeruminis*. However, Stenotrophomonas rhizophila only occurred in sample B, and the *Actinomyces* sp. was also missing from Sample B. Acinetobacter indicus was not found in Sample A but appeared in all other samples. *Aerococcus* sp. is known to be efficient at degrading hydrocarbon and has been reported in environments associated with hydrocarbon [49]. *Stenotrophomonas rhizophila* has been reported to produce novel esterases and lipases, which are top cellulolytic enzymes [50]. *Stenotrophomonas rhizophila* has been isolated from the rhizosphere of potato, deep sea invertebrates, and oilseed and known to produce secondary metabolites that possess antibiotic abilities against fungi and bacteria [51]. *S. rhizophila* showed potential to degrade keratin via keratinolysis, an enzymatic process involving sulfitolytic and proteolytic enzymes [52]. Laccase capable of degrading lignin has been reported in *Stenotrophomonas maltophilia* and, thus, *Stenotrophomonas* sp. can be inferred as a good potential for biotechnological applications [47]. *Burkholderia* sp. was also a top species. It secretes enzymes that break down cellulose, hemicellulose, lignin, and xylose and has been known to contain genes coding for catalases and peroxidases [47]. *Pseudomonas*, *Streptomyces*, and *Rhodococcus*, which were also top species, are known microbial polymer degraders [52]. The efficient breakdown of lignocellulose is a combined effort of several enzymes and, thus, it has become necessary to examine the enzymatic operation of bacterial communities [41]. For enzyme-based lignocellulosic biomass bio refineries to be more adoptable, the cost of lignocellulose-degrading enzymes, involved in converting lignocellulosic biomass to fermentable sugars and other useful products, must be significantly lowered [53].

PICRUSt provided an insight into the metabolic nature of bacteria in this particular landfill. We searched for lignocellulolytic enzymes and found that there were sequence reads that predicted some of them, and this was summarized in Figure 8. Samples A and B, despite having lower indices for richness and diversity, had higher representation of predicted genes related to lignocellulolytic enzymes. Samples A and B had 72% and 69% of the entire predictions for hydrolytic and ligninolytic enzymes involved in lignocellulose breakdown, respectively. This presents an argument that richness and diversity do not connote for higher function expressions. As waste ages in a landfill, its concentration changes as different biochemical processes occur. Thus, Samples A and B, with a longer aging period, can be said to contain more complex substances and, as a result, had more presence of hydrolytic and ligninolytic enzymes. Samples C and D, with shorter aging periods, had lower enzyme functions, with Sample D having the lowest values for all enzymes investigated. This may support findings from Deng et al. [54], who reported that the complexity of a substrate affects the way bacteria interact. While a complex substrate such as lignocellulose promotes positive interactions and synergistic growth, a labile substrate such as glucose promotes negative interactions and competition. It can be argued that the enzymes were expressed less in sites where their energy source was less. However, all samples predicted lignocellulolytic enzyme function, as there was function prediction for cellulase, xylanase, peroxidase, amylase, chitinases, esterase, and mannanase. Thus, our findings support that bacteria are involved in lignocellulolytic degradation in the landfill. The biodegradation of these waste materials being deposited in the landfill creates a biodiversity that is a source of genomic and biochemical information that can be used potentially in industry and bioremediation processes [55].

## 5. Conclusions

Bacteria in the landfill are a rich pool of lignolytic and hydrolytic diversity. This study supported our hypothesis that the Pulau Burung landfill, as in other landfill studies, is a huge depository of lignocellulolytic bacteria. The results included known members of bacterial community involved in lignin, cellulose, and hemicellulose degradation. These can be mined for use in lignocellulose-driven bio refinery and industrial processes. These findings also help to suggest possible co-cultures of bacteria, as lignin and cellulose breakdown in nature is achieved by synergistic relationship of several microorganisms and not by a single culture. The design of communities to mirror that seen in this study will lead to developing new and more effective enzyme cocktails that can be used in pre-treatment and saccharification of lignocellulose biomass into valuable products including but not limited to biofuels, chemicals, enzymes’ bio char, and additives.

## Figures and Tables

**Table 1:** Physicochemical properties of sediments’ samples collected from the Pulau Burung landfill.

Samples	pH	Temperature (°C)	Moisture Content (%)	C (%)	H (%)	N (%)
A	6.80 ± 1.13	35.00 ± 1.01	17.07 ± 4.08	7.64 ± 0.18	0.88 ± 0.17	0.30 ± 0.06
B	6.82 ± 0.12	45.00 ± 0.86	3.41 ± 1.56	13.36 ± 5.29	1.88 ± 1.19	0.75 ± 0.47
C	8.44 ± 0.34	32.00 ± 0.08	7.66 ± 2.01	0.55 ± 0.14	0.44 ± 0.13	0.14 ± 0.02
D	7.98 ± 0.48	33.00 ± 1.87	37.26 ± 5.8	17.84 ± 7.51	2.25 ± 2.16	0.67 ± 0.55

Samples were analyzed in triplicate and the data were expressed as the mean ± standard error.

**Table 2:** Diversity indices and measures of the bacterial community in the Pulau Burung landfill.

Sample	Observed OTUs	Chao1	ACE	Shannon	Simpson
A	539.79 ± 28.33 ^c^	570.02 ± 0.55 ^c^	570.84 ± 0.35 ^c^	3.52 ± 0.01 ^c^	0.92 ± 0.02 ^b^
B	512.00 ± 0.09 ^d^	538.52 ± 0.57 ^d^	543.32 ± 0.90 ^d^	4.16 ± 0.03 ^b^	0.95 ± 0.01 ^ab^
C	837.01 ± 0.14 ^b^	908.68 ± 0.56 ^b^	908.46 ± 0.48 ^b^	4.59 ± 0.07 ^a^	0.97 ± 0.02 ^a^
D	1253.63 ± 0.57 ^a^	1316.53 ± 0.49 ^a^	1314.50 ± 0.55 ^a^	4.50 ± 0.04 ^a^	0.91 ± 0.01 ^b^

Samples were analyzed in triplicate and the data were expressed as the mean. The different letters indicate significant differences at *p* < 0.05. ^a–d^ Means in the rows shows the differences in the means. The same superscripts mean they are not significantly different while different superscripts mean they are significantly different.

## Data Availability

The raw sequencing data were deposited in the NCBI short-reads archive database (Accession Number: SUB8865950).

## Supplementary Materials

The following are available online at https://www.mdpi.com/article/10.3390/life11060493/s1, Figure S1: 16S Amplicon sequencing analysis pipeline, Figure S2: Rarefaction curve of bacterial community showing the level of saturation in the bacterial community, Table S1: Top 20 species in the bacteria community.
