# Supplementary material for: Bacterial Diversity and Community Structure of a Municipal Solid Waste Landfill: A Source of Lignocellulolytic Potential
Source: Life (Basel). 2021 May 28;11(6):493. doi: 10.3390/life11060493 (PMC8228822; doi:10.3390/life11060493)
Supplement: Supplementary file 1 [file life-11-00493-s001.zip › life-1184506-supplementary.pdf]

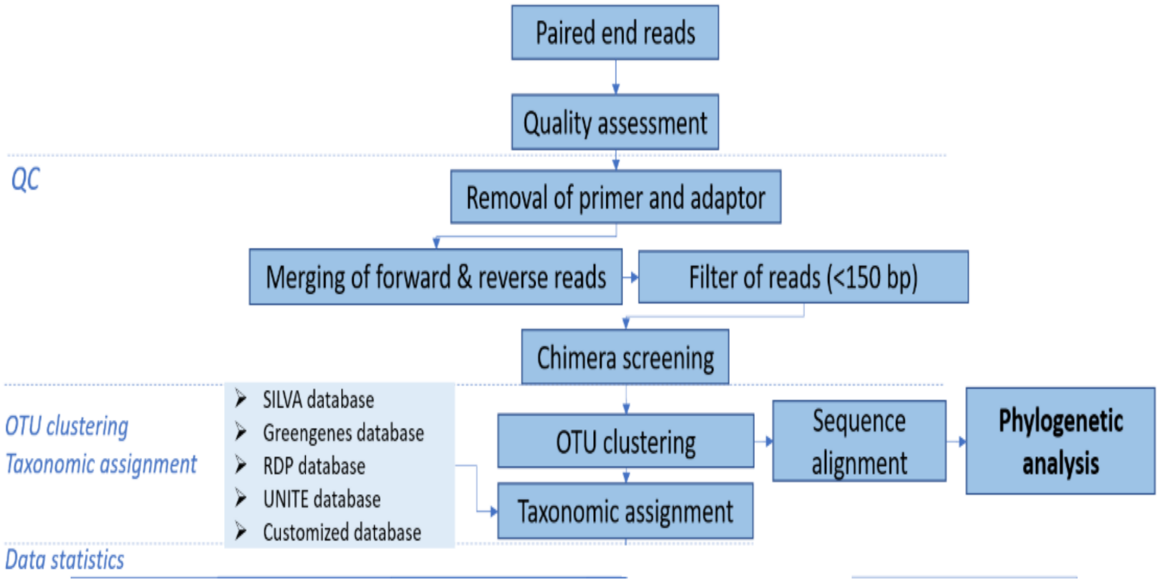

Figure S1. 16S Amplicon sequencing analysis pipeline.

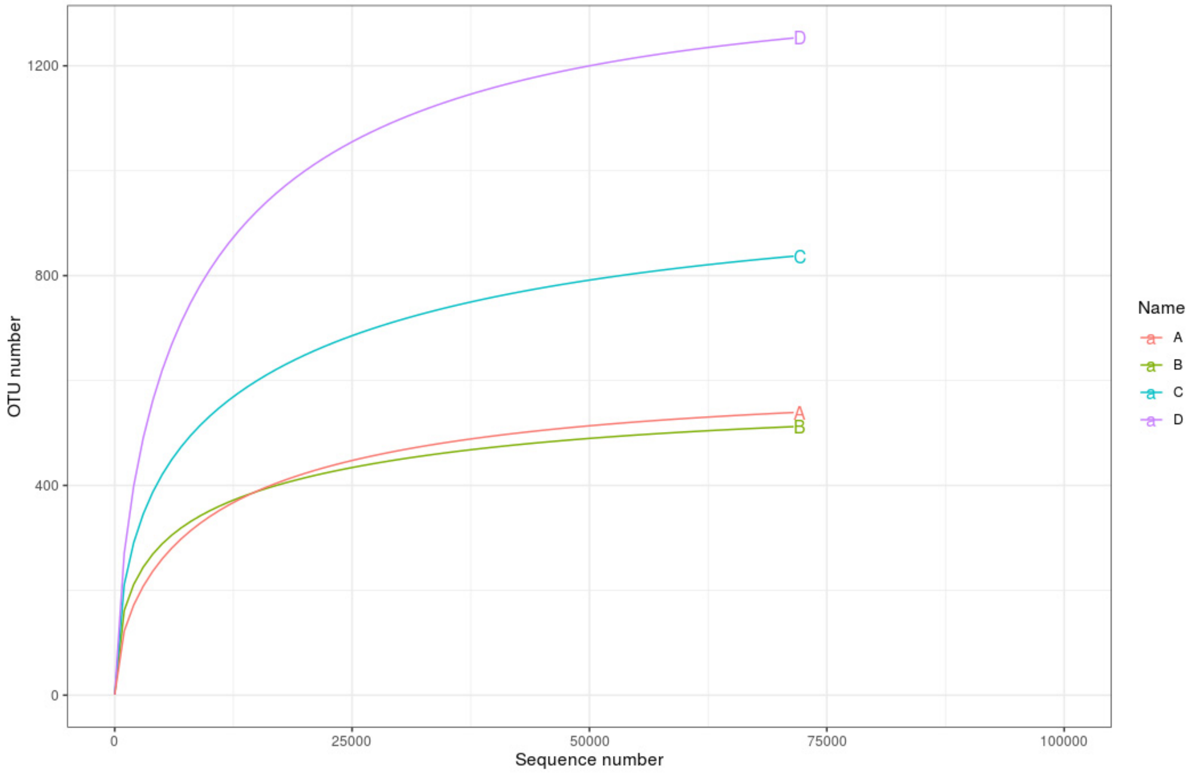

Figure S2. Rarefaction curve of bacterial community showing the level of saturation in the bacterial community.

Table S1. Top 20 species in the bacteria community

	s__Aerococcus_urinaeequi	s__Stenotrophomonas_rhizophila	s__Sporosarcina_psychrophila	s__Pseudoxanthomonas_taiwanensis	s__Corynebacterium_vitaeruminis	s__Enterococcus_italicus	s__Acinetobacter_indicus	s__uncultured_bacterium
A	0.000218	0.354962	0.206401	4.84E-05	9.68E-05	0.049803	0	0.000169
B	0.005303	0	0.000216	0.133925	0.007954	0.00037	6.17E-05	0.066223
C	0.221405	0	0.119788	0.0013	0.054669	0.060362	0.087345	0.032313
D	0.48446	0	2.38E-05	7.13E-05	0.065608	0.012342	0.01724	0.001665

	s__Sanguibacter_keddieii	s__Actinomyces_sp.	s__Weissella_paramesenteroides	s__Lactobacillus_amylovorus	s__Bavariicoccus_seileri	s__Enterococcus_cecorum
A	0.055517	0.083481	0.02271	0.018667	0.06186	0.006392
B	9.25E-05	0	0.001172	9.25E-05	9.25E-05	0.000185
C	0.040891	0.000156	0.005589	0.015961	0.000104	0.040605
D	0.001118	0.000214	0.043659	0.030176	0.001783	0.008989

	s__uncultured_bacterium.1	s__uncultured_bacterium.2	s__Pseudoxanthomonas_suwonensis	s__Chelativorans_composti	s__uncultured_Luteimonas	s__Clostridium_sp.
A	0	0.000169	0	0	0	0
B	0.047355	0.042576	0.03453	0.03863	0.032649	0
C	0	0.001248	0.004835	0	2.6E-05	0.025398
D	0.000238	0.000999	0.000523	9.51E-05	0	0.006944
